# Role of minimally invasive surgery in the treatment of gallbladder metastatic melanoma. A review of the literature and a case report

**DOI:** 10.1002/cnr2.1549

**Published:** 2022-01-03

**Authors:** Petros Ioannis Bangeas, Alexandra Bekiaridou, Alexandros Tsolakidis, Kostantinos Georgios Efthymiadis, Kostantinos Drevelegkas, Dimitris Giakoustidis, Alexandros Giakoustidis, Petros Alexidis, Vassileios Nikolaos Papadopoulos

**Affiliations:** ^1^ 1st University Department of Surgery School of Medicine, Aristotle University of Thessaloniki Thessaloniki Greece; ^2^ Radiology Department General Clinic of Thessaloniki Thessaloniki Greece; ^3^ 1st University Oncology Department School of Medicine, Aristotle University of Thessaloniki Thessaloniki Greece; ^4^ Department of Radiation Oncology Papageorgiou Hospital Thessaloniki Greece

**Keywords:** gallbladder, intramucosal metastasis, metastatic melanoma

## Abstract

**Background:**

Primary and secondary gallbladder melanomas are rare, and only 58 cases have been reported in scientific literature to date. This paper aimed to explore the role of minimally invasive surgery in the management of gallbladder metastatic melanomas.

**Case:**

Herein, we present the case of a 68‐year‐old man with metastatic gallbladder melanoma who was treated with laparoscopic cholecystectomy. Our case management was then compared with that of other cases reported in the literature.

**Conclusion:**

Currently, metastatic melanomas can be considered as a potentially curable disease. Palliation of symptoms and fast recovery following minimally invasive procedures could be beneficial for these patients. Particularly, laparoscopic procedures appear to prolong the survival of gallbladder melanoma patients.

## 1 INTRODUCTION

Melanoma develops from melanocytes and is present mainly in the skin, eyes, and gastrointestinal mucosa. Most metastases originate from cutaneous lesions. However, mucosal, meningeal, or ocular sites of origin have been reported in the literature. The majority of patients have localized disease at the time of diagnosis and are treated with local excision. However, several patients develop distant metastases.^1^ Primary malignant melanoma of the gallbladder (MMG) is theoretically possible. The presence of melanoma cells in the gallbladder can be easily explained. During embryogenesis, melanin‐producing cells migrate from the neural crest to the endoderm.^2^ Primary MMG is a rare entity, with the first case reported in 1907; in 1957, Walsh reported the first histologically proven case of primary MMG.

Cutaneous melanoma metastases can affect all organs of the human body. The most common sites are the lymph nodes, lungs, liver, and brain. Although isolated gallbladder metastases are extremely rare, almost 50% of all secondary gallbladder metastases are attributed to melanoma, as reported by an autopsy series.^3^

Gallbladder lesions are usually asymptomatic, but in some cases, no specific symptoms, such as right upper quadrant pain, can be present.^4^

Ultrasonography (U/S) usually has atypical features, and in most cases, a hyperechoic lesion is observed. Additional information can be gotten from computed tomography (CT) and magnetic resonance imaging (MRI).^5^

MMG is associated with a very poor prognosis, and the optimal treatment strategy remains ambiguous.^1^

However, the indication of laparoscopic surgery remains unclear. Laparoscopic cholecystectomy (LC) is a feasible and efficient technique for metastasectomy of melanoma. LC appears to be the safest option because it is associated with less intraoperative blood loss, low analgesic requirement, shorter length of hospital stay, and brief convalescence. All of the above advocate for the desired result to increase the quality of life of each patient. On the other hand, the open approach is required in the case of multiple metastases as it provides a broader view of the abdomen. Nevertheless, if metastasis is observed to be limited, after radiological examination, there will be no indication for open surgery.

Herein, we presented the case of a patient with MMG detected during typical follow‐up examinations. In addition, a review with a statistical analysis of all the cases reported in the past, discussing the most common clinical features as well as the role of laparoscopic surgery as a therapeutic option in the management of the disease was performed.

## 2 MATERIALS AND METHODS

**FIGURE 1:**
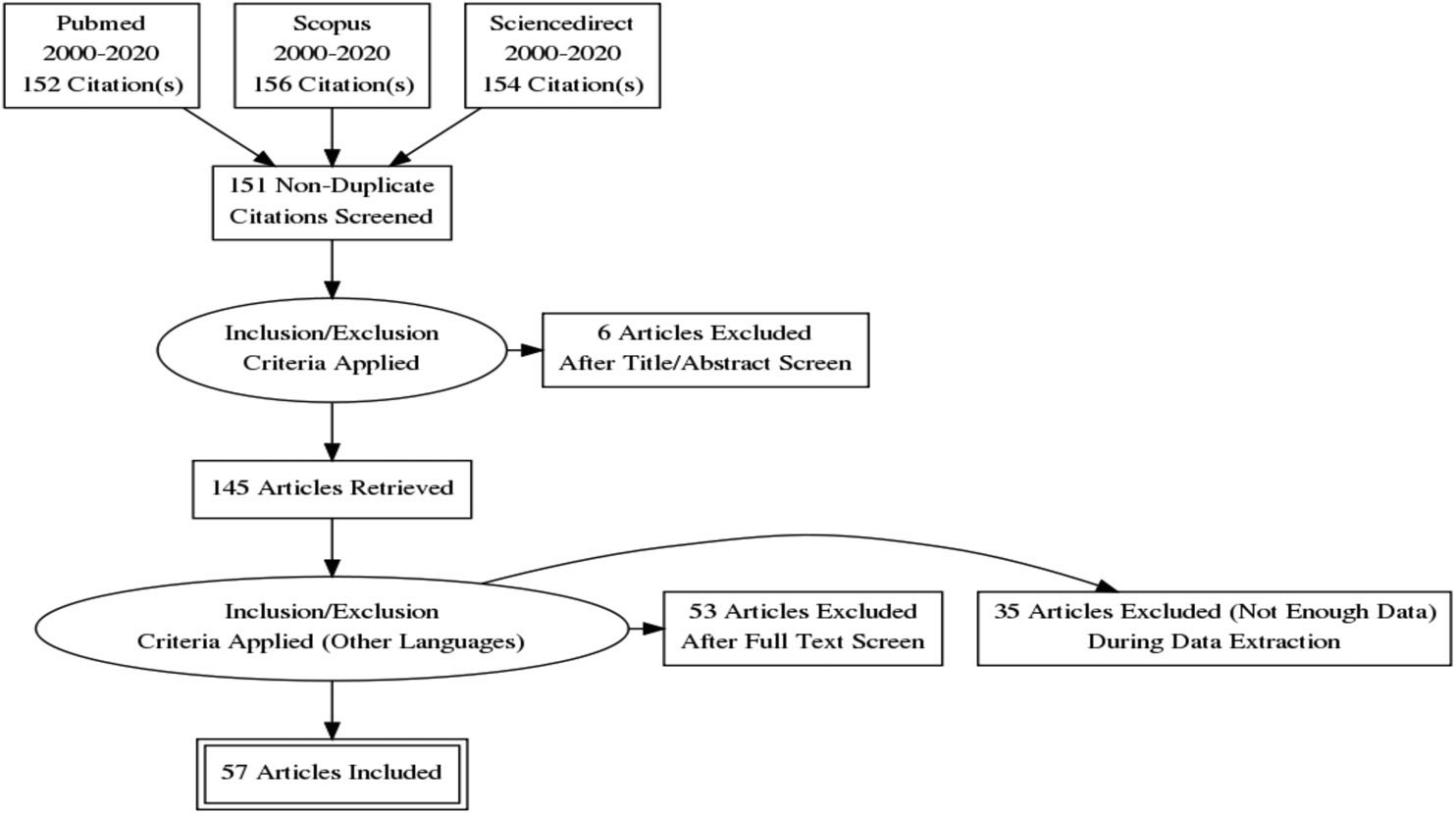
Prisma chart

A literature review was performed using PubMed, Scopus, and Science Direct. The search terms employed were “gallbladder melanoma” and “gallbladder metastatic melanoma”. Since 1955, when the first description of MMG was reported by Thayer et al., 151 articles have been published (Figure 1).

Among these, 145 well‐documented papers were identified. There were no restrictions on the age of the articles included in this review.

Ninety‐two articles were in English, while 53 were in other languages. All these studies were carefully studied. In the final assessment, we included only full texts, case reports, and case series articles. We finally selected 58 cases and a database with the patients' characteristics was created. The database included sex, age, primary tumor location, symptoms, symptom duration, tumor size, diagnostic methods, treatment, metastasis, and follow‐up. The cases that fulfilled at least eight of these 10 criteria were included in the statistical analysis. One additional case was added from the clinical experience of the authors of this article. Thus, a total of 58 cases were included in the statistical analysis. After obtaining ethical approval and participant consent, personal data were removed, and all clinical data were collected.

Descriptive statistics were used to appropriately express the results. Means, medians, and SD were used for continuous variables and frequencies for categorical variables.

The Spearman test was used to calculate the correlation between histological infiltration and age. An independent samples *t*‐test was used to calculate the distribution of age among males and females. The histological layer infiltration of the tumor of male versus female subjects was compared using the Mann–Whitney *U*‐test. The Kruskal‐Wallis test was also used to calculate the distribution of layer infiltration across categories of survival in months. For layer infiltration and the type of procedure, the Kruskal‐Wallis test was calculated for this correlation. Statistical significance was set at *p* < .05. Statistical analysis was performed using SPSS version 25 (SPSS Inc., Chicago, IL, USA).

## 3 CASE REPORT

Our case was a 68‐year‐old Caucasian man with a history of hypertension. The patient was brought to the emergency department due to epilepsy. A CT scan revealed a brain tumor in the left temporal region. The patient was urgently taken to the operating theater due to hydrocephaly. The final pathological specimen of the brain tumor revealed a metastatic melanoma.

**FIGURE 2:**
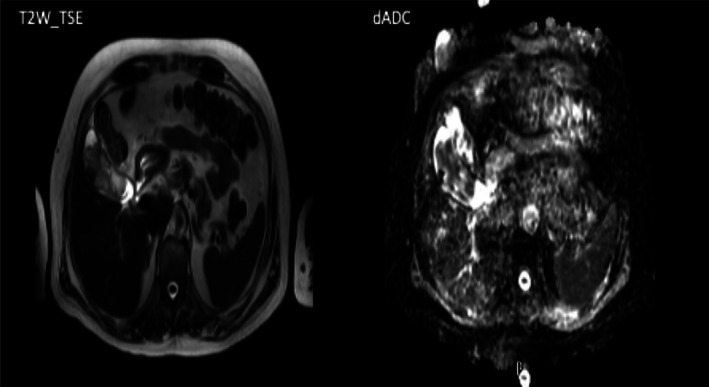
Apparent diffusion coefficient sequence of neoplasm showed low signal while in the diffusion‐weighted imaging intermediate signal intensity was revealed

Complete skin examination did not reveal any primary skin lesions. The history of the patient revealed that, 2 years ago, a scabby lesion was discovered on his right ear. The lesion was subsequently widely excised. The lesion was initially thought to be a basal cell carcinoma. After repetitive consultations and pathological specimen examination, it was revealed that the lesion was a melanoma, which was retrospectively staged as T3N0M0, stage IIa. Thus, the patient was put on observation. After the brain surgery, the patient underwent 10 fractions of whole‐brain radiotherapy and received 30 Gy of radiation. Initial postoperative MRI of the brain did not show any recurrence, while upper abdomen MRI revealed a large lesion with perspiration arterial strain, without extracorporeal infiltration. The apparent diffusion coefficient sequence of the neoplasm showed low signal intensity, while the diffusion‐weighted imaging, showed an intermediate signal intensity (Figure 2). Given the progression, the oncologist ordered a positron emission tomography (PET) scan, which revealed metastatic spread to the gallbladder (SUVmax 9,9).

As such, following the multidisciplinary team's discussion, according to the European society for medical oncology guidelines and due to the patient's good performance status, LC was decided. Patient blood testing revealed *a white cell count* of 5.89 × 10^9^/L, *hemoglobin* 13.1, *AST* 12 U/L, *ALT* 14 U/L, *BIL* 0,34, and *GGT* 57 U/L.

LC was performed under general anesthesia. The postoperative period was uneventful, and the patient was discharged 2 days later in good condition. After the final specimen examination, the results were positive for melanoma, while immunohistochemical staining showed strong positivity for HMB‐45, MART‐1, and S‐100 protein. Molecular testing revealed no *BRAF* mutations.

According to the oncological multidisciplinary team recommendations, further treatment for resected stage IV metastatic melanoma was decided after the patient was discharged from the hospital. He received nivolumab 240 mg Q2W for 6 months (12 cycles). Restaging imaging at 12 months with CT CAP and brain MRI revealed CNS recurrence of the resected left temporal lobe lesion, and stereotactic radiosurgery with γ Knife was decided in the MDT meeting, which was performed successfully. He continued treatment with nivolumab and is currently still on treatment. He has received 20 cycles and no evidence of metastatic disease was noted at his last restaging visit until today.

## 4 RESULTS

The characteristics of MMGs were determined based on sex, age, primary localization of the tumor, other metastases, major symptoms, infiltration histology layer, type of procedure, and survival. Regarding sex, 67.2% of the patients were male (39 patients), whereas 32.8% were female (19 patients). The ratio between men and women was 3:1, suggesting a male predominance in the reported MMG population.^6^, ^7^, ^8^, ^9^, ^10^, ^11^, ^12^, ^13^, ^14^, ^15^, ^16^, ^17^, ^18^, ^19^, ^20^, ^21^, ^22^, ^23^, ^24^, ^25^, ^26^, ^27^, ^28^, ^29^, ^30^, ^31^, ^32^, ^33^, ^34^, ^35^, ^36^, ^37^, ^38^, ^39^, ^40^, ^41^, ^42^, ^43^

**FIGURE 3:**
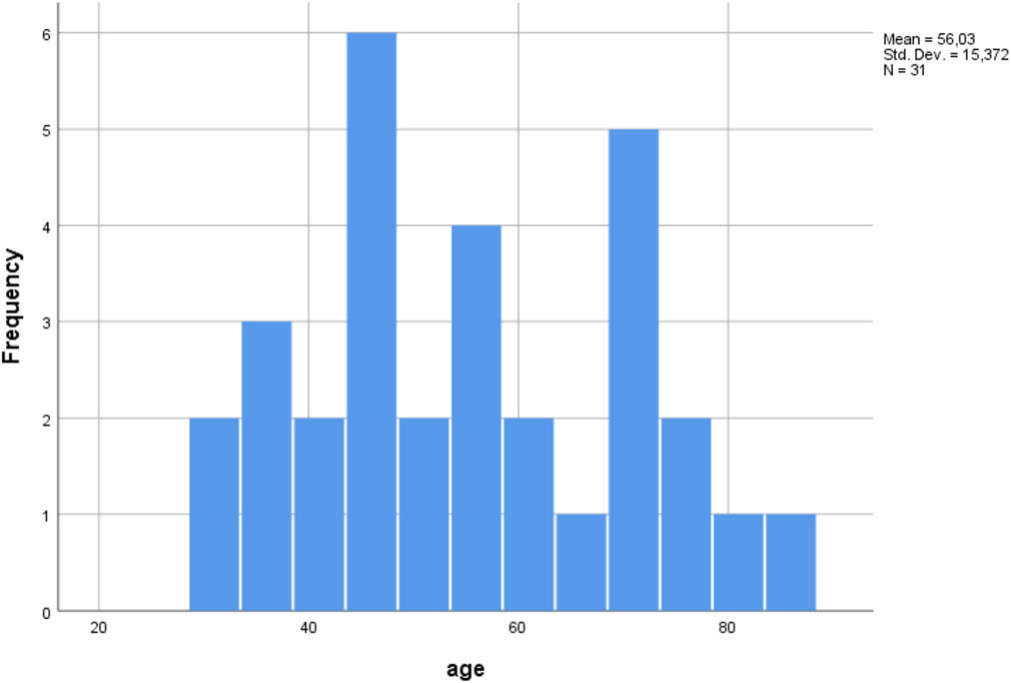
Age distribution among genders

The age distribution is shown in Figure 3, from which it is concluded that MMG most frequently appeared in the age ranges of 36–41, 54–59, and 72–77 years. The mean age of the 58 patients (100%) was 55 ± 14.82 years, ranging from 30 to 86 years. The distribution of age among male and female patients was compared, and it was found that the age at which the tumor appeared did not differ statistically between males and females (55.95  ±  15.587 and 53.63  ±  14.241 years, respectively, *p*  =  0.587).

As far as the primary location of melanoma is concerned, in our database, the most common locations, were legs (nine patients, 15.52%), back (seven patients, 12.07%), pleura (six patients, 10.34%), shoulder (six patients, 10.34%), face (five patients, 8.62%), arm (three patients, 5.17%), abdomen (three patients, 5.17%), eyes (two patients, 3.45%), and neck (one patient, 1.72%). There are also cases reported in the literature, in which MMGs occurred without the detection of the primary melanoma (16 patients, 27.59%).

**FIGURE 4:**
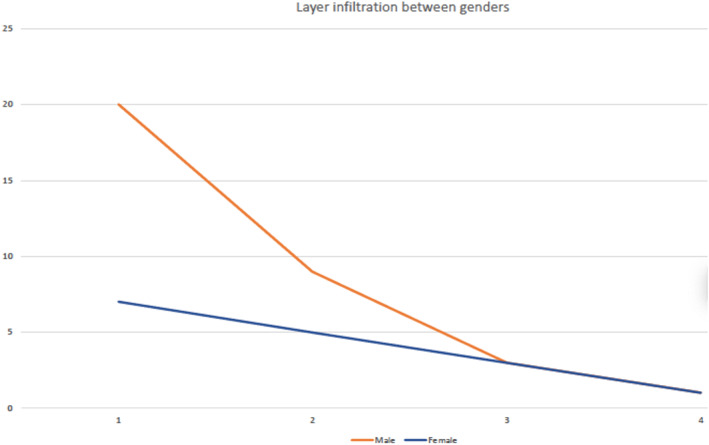
Layer infiltration among genders (X‐axis: 1, Intramucosal, 2, Muscolaris propria, 3, Muscle layers, 4, All layers. Y‐axis: Percentage number of patients)

Histology layer infiltration ranged from intramucosal infiltration (27 patients, 46.6%), mucosal and lamina propria infiltration (14 patients, 24.1%), muscularis propria infiltration (six patients, 10.3%), and infiltration of all layers (two patients, 3.4%). Statistical analysis showed that there was no statistically significant correlation between the degree of tumor infiltration and age (*p*  = 0.237, *r*  =  − 0.172). Comparison of the infiltration degree of male and female subjects proved that there was also no significant difference (U  =  211.50, *p*  =  0.213) between genders (Figure 4). The layer infiltration was also compared with the outcome of the disease in each patient, by assessing the months of survival (*p* = 0,101).

**FIGURE 5:**
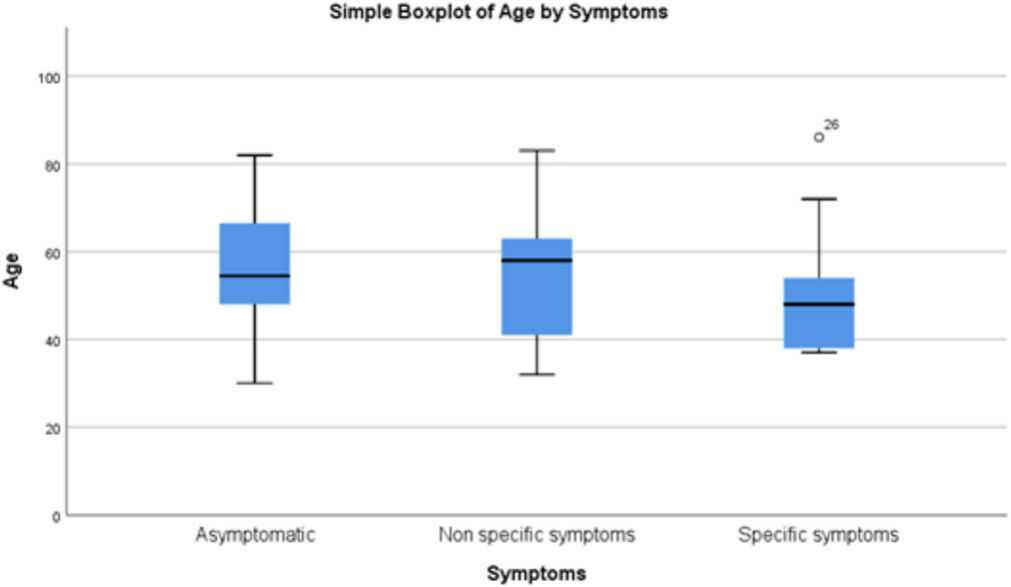
Symptomatology and age correlation with the total number (26) of symptomatic patients in the last column

The symptomatology of MMG was also evaluated. Symptoms were characterized as nonspecific, including lack of appetite, fatigue, progressive weakness, chest or back pain (26 patients, 44.83%), and as specific symptoms, including typical acute cholecystitis (nine patients, 15.5%). There were also 23 patients (39.65%) who presented without symptoms (Figure 5).

Diagnostic tests required for the diagnosis of MMG were also considered. CT, MRI, laboratory tests, and PET seemed to be the most common ways to diagnose MMG or exclude other similar conditions. For the histopathological and immunohistochemical examination of MMGs, among the 47 (81.03%) cases with available data, 44 (93.62%) were positive for HMB45 monoclonal antibody, S100, and Melan‐A indexes. Nonetheless, there was no evidence from the other 11 patients (18.96%).

Surgical excision is the primary treatment for patients with MMG. Concerning the type of procedure, open cholecystectomy was performed in 29(50%) out of 58 patients whereas LC was performed in 19(32.76%) out of 58 patients. Ten (17.24%) patients out of 58 patients did not undergo any surgery. Duration of hospitalization was provided only for 14 cases (24.14%), giving this analysis low credibility. However, the median hospitalization duration was 4 days, ranging from 2 to 10 days, depending on the patient's performance status. A Kruskal‐Wallis test was performed to determine whether there was any correlation between layer infiltration and type of procedure. However, no significant results were found (Sig = 0.758, H = 0.554, *p* = .05). There was also no significant correlation between the type of procedure and age (Sig = 0.454, H = 1.578, *p* = .05). Comparing the type of procedure and mean survival, the Kruskal‐Wallis test between three therapeutic choices (Open, Laparoscopic, no surgery) showed no significant difference (Sig = 0.069, H = 5.358, *p* = .05).

The follow‐up period was mentioned in all 58(100%) patients. Forty‐two (72.41%) patients died during the follow‐up period. The median survival period was 13 ± 16.27 months, ranging from 0 to 59 months. Nine (15.52%) patients were < 6 months of age. During the follow‐up period, recurrence of melanoma in other sites developed in eight (13.79%) patients.

## 5 DISCUSSION

### 5.1 Epidemiology

Primary tumors commonly occur on the skin (90%) and are strongly associated with excessive sunlight exposure. However, they can also develop from other tissues containing melanocytes, such as the meninges, GI, mucosa, and eyes.^44^ Cutaneous melanoma can metastasize to almost any tissue. The most frequent sites of metastasis are the liver, lungs, and brain.^2^, ^45^ Spread to the gastrointestinal tract is found in 2%–4% of patients.^3^ Mostly, it affects the small bowel (35%–67%), colon (9%–15%), and stomach (5%–7%).^2^, ^4^ Although metastasis due to melanoma is the most common metastatic tumor of the gallbladder, it is considered extremely rare. Primary MMG may be derived from melanocytes that migrate from the neural crest to the endodermal tissue during embryogenesis.^5^ The most commonly accepted theory regarding the dissemination of the gallbladder states that it occurs via the bloodstream. Another theory supports that it occurs due to the implantation of metastatic cells into the mucosa. These cancer cells may be derived from microscopic metastatic foci in the liver and migrate to the gallbladder through bile flow.^2^ This theory could also explain the common synchronous dissemination of the gastrointestinal tract.^4^, ^5^ MMG has been associated with diffuse metastatic disease in most cases, with only a few cases describing MMG as the sole site of metastasis upon diagnosis.

MMGs have been discovered in post‐mortem examinations in 15% of melanoma patients.^45^, ^46^ However, there are only 58 reported cases of patients, and the silent nature of the disease may contribute to this phenomenon.^2^, ^45^, ^46^, ^47^

From an epidemiological standpoint, based on available data, the male‐to‐female ratio was 3:1 (39 males vs. 19 females), and the median patient age was 54 ± 14.82 years.

### 5.2 Clinical features

The majority of melanoma metastases cases are asymptomatic, while the tumor is usually discovered in post‐mortem examinations or has been found incidentally during the routine follow‐up period. When symptomatology is present, the clinical features are variable, mimicking acute cholecystitis with sudden right upper quadrant abdominal pain. Secondary symptoms may include hemobilia, melena, nausea, and jaundice, mostly due to obstruction of the common bile duct.^1^, ^5^, ^47^, ^48^

The predominant symptoms of symptomatic tumors are epigastric pain after eating and fever. Physical examination revealed severe tenderness in the right upper quadrant of the abdomen with a nonpalpable gallbladder.

### 5.3 Diagnosis

The extreme rarity of the disease is responsible for the absence of standard diagnostic and therapeutic guidelines. First, as highlighted above, physical examination was due to some other indications. Laboratory tests were usually normal; in some cases, they showed an elevation of white blood cell count with neutrophilia and an increased total serum bilirubin level. Liver function studies ranged from normal, in some cases, moderately increased to highly increase due to severe liver dysfunction.

The arrows in the diagnosis quiver were U/S and CT. U/S is the examination of choice for abdominal assessment. In this case, a single polypoid mass was present, but generally, U/S did not demonstrate lithiasis but a single or multiple polypoid, hyperechoic mass with minimal to absent acoustic shadowing. In contrast to gallbladder cancer, MMG does not seem to be associated with cholelithiasis.^2^ Dilation of biliary ducts may also occur, and more often, dilation of the common hepatic duct. On CT scan, lesions showed enhancement after administration of an intravenous contrast agent and appear as isodense to hyperdense (compared to muscle density).

On MRI, lesions typically show T1 signal hyperintensity and T2 signal hypointensity. Lesions may occur while enhancing the biliary and gallbladder mucosa. The administration of gadolinium contrast is not usually helpful. Magnetic resonance cholangiopancreatography (MRCP) and endoscopic retrograde CP exhibit polypoid filling defects or irregular narrowing of the extrahepatic duct.^44^

PET scans can reliably help visualize malignant lesions in structures that are still macroscopically normal. Melanoma was one of the first indications for Medicare‐approved coverage for PET. While PET is on routine follow‐up imaging, it can also be a useful tool to verify the hypothesis of melanoma metastasis.

All of the above findings help in the differential diagnosis of the mass, which includes cholesterol polyps, inflammatory polyps, adenomyomas, and carcinoma of the gallbladder.^4^

Although all these tests indicate gallbladder disease, it is only the histopathologic examination that can diagnose and discriminate MMG correctly from other gallbladder tumors. Histopathologically, tumor cells were atypical with a rounded nucleus, oval, or even polylobed and nucleated with an eosinophilic cytoplasm with brownish pigments. In some cases, the cells appeared hyperchromatic with inconspicuous nucleoli. There was also the occurrence of mitoses. Despite the proliferation of immunochemistry markers, S‐100 remains the most sensitive marker for melanocytic lesions. Marker S100 in combination with HMB45 and Melan‐A demonstrated relatively good specificity for the metastatic melanocytic nature of the lesion. The role of the HMB45 marker is questionable in the literature, it has been found in metastatic diseases, while it has disappeared in rapidly growing primary tumors. According to Skelton et al., a positive reaction with HMB45 indicates active melanosome formation and, thus, melanocytic differentiation.

Faced with the suspicion of vesicular metastasis of cutaneous melanoma, a complementary immunohistochemical (p16, desmin, and BRAFV600E) and molecular (BRAF gene sequencing, in search of mutations) study was performed and was compatible with a secondary location. BRAF mutation testing has become a priority for determining oncologist choices. Generally, the detection of BRAF mutations is an excellent screening method, but the possibility of false negatives has also been reported.^49^

### 5.4 Pathology

Mucosal melanomas can occur in a variety of unusual sites. Resection of these lesions depends on the primary location and the performance status of the patients. Our data support that MMG should be excised when diagnosed. Two types of excision recommended in the literature are: Open and endoscopic. Most cases were treated with classic open surgical excision (45.2%). In cases of diffuse disease, open surgical excision is advantageous in avoiding clinical manifestations of the disease and preventing further dissemination.^2^, ^50^, ^51^ In these cases, open cholecystectomy followed by partial V‐segmentectomy is performed. Although an open approach is often preferable,^3^ laparoscopic dissection, when executed by an experienced surgeon, is an adequate and highly efficient method. Statistically, there was no significant difference between histological layer infiltration and the preferred procedure (Sig = 0.132, H = 5.73). In addition, there was no significant correlation between the type of procedure and the patient's age (Sig = 0.146, H = 3.85).

One of the major risks of the laparoscopic approach is intraperitoneal bile spillage from the gallbladder. Disruption of bile spillage by the gallbladder seems to be the main cause of port‐site metastases or peritoneal metastases, especially in patients with pT1 tumors. However, this precaution does not always exclude an intraperitoneal seeding event. An isolation bag is useful to remove the resected specimen from the abdominal cavity, and this method usually does not cause peritoneal dissemination in the peritoneal cavity. Gentle manipulation, avoidance of perforation, and use of a retrieval bag for the removal of the gallbladder should be practiced helping minimize the chance of mechanical exfoliation or implantation of malignant cells during LC.^52^, ^53^, ^54^, ^55^, ^56^

Lymphadenectomy is performed in primary MMG, while metastatic cases are not needed when the lesion is intraluminal. In cases of doubt between primary and metastatic MMG, lymphadenectomy was also performed with the LC. Laparoscopic lymphadenectomy of the hepatoduodenal ligament is a technically demanding procedure that can be performed by an experienced laparoscopic surgeon with a remarkably low complication rate.^54^

Comparing the type of procedure and mean survival, laparoscopic surgery has a mean survival of 13.2 months, while open cholecystectomy has a mean survival of 8.95 months. Nevertheless, there were no significant differences between the therapeutic strategies (Sig = 0.327, H = 2.23). In the histogram, the laparoscopic and open procedures showed a normal distribution. In contrast, the choice of not proceeding with surgery shows a negative skewness. The above data suggest that conservative manipulation strategies (without surgery) were the treatment of choice for older patients or patients with poor prognoses.

Recent data on metastatic disease survival following treatment with checkpoint inhibitors for *BRAF* wild‐type melanoma show that nearly half of the patients were still alive 5 years after treatment initiation.^52^, ^53^

Dual checkpoint inhibition with the anti‐PD1 antibody nivolumab combined with the anti‐CTLA1 antibody ipilimumab compared with ipilimumab monotherapy showed a remarkable 5‐year overall survival rate of 52% versus 26% (HR = 0.52, 95% CI = 0.42–0.64). Moreover, nivolumab monotherapy compared to ipilimumab monotherapy also achieved a high overall 5‐year survival rate of 44% versus 26% (HR = 0.63, 95% CI = 0.52–0.76).^52^

Likewise, anti‐PD1 monotherapy with pembrolizumab compared to ipilimumab also offers improved survival with a median OS of 33 months versus 16 months, and a 5‐year OS of 39% versus 31% (HR 0.73, 95% CI 0.61–0.88).^53^

These groundbreaking results underline the fact that metastatic melanoma can be considered a potentially curable disease, and we consider complete resection of isolated lesions of oligometastatic disease to be of great importance in the era of immunotherapy, possibly enhancing these already remarkable long‐term survival outcomes.

## 6 CONCLUSION

In the present case of a 68‐year‐old man, the decision was to perform laparoscopic resection of the gallbladder. Regarding treatment, other retrospective studies in the literature confirmed an improvement in the 5‐year overall survival rates in patients with stage IV melanoma who underwent metastasectomy, compared to patients who were ineligible for resection. However, these reports were obtained from specialized single institutions.

Despite the technical challenges, LC was considered the treatment of choice, as the lesion to be resected, was restricted only to the gallbladder and with no other obvious site of metastasis. Local nodal dissection was not performed because the tumor was intraluminal.^50^, ^51^

Metastasectomy combined with immunotherapy of melanoma offers a strong advantage to these patients, thus, prolonging their survival. Proceeding with LC offered the advantage of quick recovery, as the patient continued therapy immediately with an immunotherapeutic agent. The 12‐month survival time of the patient was reported, which is comparable with most of the cases in the published literature, as well as the fact that he has remained recurrence‐free. This demonstrates the full potential of laparoscopic surgery as a modern and completely safe alternative to the norm.^57^, ^58^, ^59^ In cases with isolated and resectable primary and metastatic gallbladder melanoma, LC has also been proposed as adequate treatment.

The scarcity of reported cases does not allow us to suggest the laparoscopic approach as the procedure of choice. However, palliation of symptoms and fast recovery after minimally invasive procedures could be beneficial for patients.^51^ While there is no statistical evidence from the literature analysis, to advocate the open approach.

## CONFLICT OF INTEREST

The authors whose names are listed immediately below certify that have no affiliations or involvement in any organization or entity with any financial interest in the subject matter or material discussed in this manuscript. Petros Bangeas, Alexandra Bekiaridou, Alexandros Tsolakidis, Kostantinos Georgios Efthymiadis, Kostantinos Drevelegkas, Dimitrios Giakoustidis, Alexandros Giakoustidis, Petros Alexidis and Vassileios Nikolaos Papadopoulos.

## AUTHOR CONTRIBUTIONS

**Petros Ioannis Bangeas:** Conceptualization (lead); data curation (lead); formal analysis (lead); funding acquisition (lead); investigation (lead); methodology (lead); project administration (lead); resources (lead); software (lead); supervision (lead); validation (lead); visualization (lead); writing – original draft (lead); writing – review and editing (lead). **Alexandra Bekiaridou:** Formal analysis (equal); methodology (equal); software (equal); writing – original draft (equal); writing – review and editing (equal). **Alexandros Tsolakidis:** Investigation (supporting). **Kostantinos Georgios Efthymiadis:** Investigation (supporting). **Kostantinos Drevelegkas:** Data curation (supporting); formal analysis (supporting); investigation (supporting). **Dimitris Giakoustidis:** Supervision (supporting). **Alexandros Giakoustidis:** Supervision (supporting); writing – review and editing (supporting). **Alexidis Petros:** Methodology (supporting). **Vassileios Nikolaos Papadopoulos:** Project administration (equal); supervision (equal); visualization (equal); writing – review and editing (equal).

## ETHICAL STATEMENT

P.I.B, A.B, A.T, K.G.E, K.D, D.G, A.G, P.A and V.N.P have nothing to disclosure. Authors also confirmed that all procedures followed, were in accordance with the ethical standards with the Helsinki declaration of 1975, as revised in 2000. Inform consent was obtained from the patient for being include in our study.

## Data Availability

Data sharing is not applicable to this article as no new data were created or analyzed in this study.
